# Advanced diffusion-relaxation imaging for tumoral differentiation and metastasis prediction in oral tongue cancer

**DOI:** 10.1186/s41747-025-00639-1

**Published:** 2025-10-08

**Authors:** Siyu Li, Wentao Hu, Gongxin Yang, Xiaofeng Zheng, Yifeng Huang, Dongmei Wu, Yingwei Wu, Yongming Dai

**Affiliations:** 1 Department of Radiology, Shanghai Ninth People’s Hospital, Shanghai Jiao Tong University School of Medicine, Shanghai, China; 2 Department of Radiology, Renji Hospital, Shanghai Jiao Tong University School of Medicine, Shanghai, China; 3 Department of Oral Pathology, Shanghai Ninth People’s Hospital, College of Stomatology, Shanghai Jiao Tong University School of Medicine, Shanghai, China; 4 Department of First Dental Clinic, Shanghai Ninth People’s Hospital, Shanghai Jiao Tong University School of Medicine, Shanghai Research Institute of Stomatology, Shanghai, China; 5 Shanghai Key Laboratory of Magnetic Resonance, East China Normal University, Shanghai, China; 6 School of Biomedical Engineering & State Key Laboratory of Advanced Medical Materials and Devices, ShanghaiTech University, Shanghai, China

**Keywords:** Carcinoma (squamous cell), Diffusion magnetic resonance imaging, Neoplasm metastasis, Perineural invasion, Tongue neoplasms

## Abstract

**Background:**

To determine the feasibility of diffusion-relaxation correlation spectroscopic imaging in identifying tumoral differentiation profile and predicting cervical lymph node metastasis (CLNM) in oral tongue squamous cell carcinoma (OTSCC).

**Materials and methods:**

This prospective study enrolled fifty-seven OTSCC patients who underwent preoperative head and neck magnetic resonance imaging (MRI). Scans with multi *b*-values (0–1500 s/mm^2^) and multi-echo times (7–150 ms) were performed to generate normalized diffusion-T2 spectra. Tumor maximal diameter and depth of invasion were measured. Tumors were segmented into five compartments (*V*_A_ to *V*_E_) with metrics compared across normal controls, CLNM-, and CLNM+ groups. Pathological parameters such as tumor-stroma ratio (TSR), perineural invasion, Ki-67, tumor p53 protein, and cyclin-dependent kinase inhibitor p16 were evaluated. Correlations between MRI metrics and pathological parameters were assessed. Predictors of CLNM+ were identified using logistic regression analysis, and the predictive performance was evaluated using receiver operating characteristic analysis.

**Results:**

Thirty-four patients were assigned to the CLNM+ group and 23 to the CLNM- group. CLNM+ patients showed larger tumor maximal diameters, deeper invasion, increased *V*_B_ and *V*_D_, and decreased *V*_A_ compared to CLNM- patients. *V*_B_ exhibited strong positive correlations with perineural invasion and depth of invasion, while *V*_D_ correlated positively with TSR. Moreover, *V*_B_ and depth of invasion were independent prognostic factors for CLNM+, and their combined model achieved the highest predictive performance.

**Conclusion:**

Diffusion-relaxation correlation spectroscopic imaging marked a significant advancement in the diagnostic and prognostic assessment of OTSCC, offering detailed tumor characterization and improving CLNM+ prediction, with great potential for accurate and non-invasive evaluation.

**Relevance statement:**

Diffusion-relaxation correlation spectroscopic imaging metrics (*V*_B_ and *V*_D_) characterized tumor heterogeneity and correlated with pathological biomarkers, making it a promising non-invasive tool for enhancing preoperative decisions and reducing unnecessary lymph node dissections in clinical workflows.

**Key Points:**

- Tumoral components and heterogeneity of oral tongue cancer were investigated on MRI.
- Advanced diffusion-relaxation imaging delineated the tumoral differential profile and predicted metastasis.
- We provided a non-invasive tool for preoperative decision-making in clinical workflows.

**Graphical Abstract:**

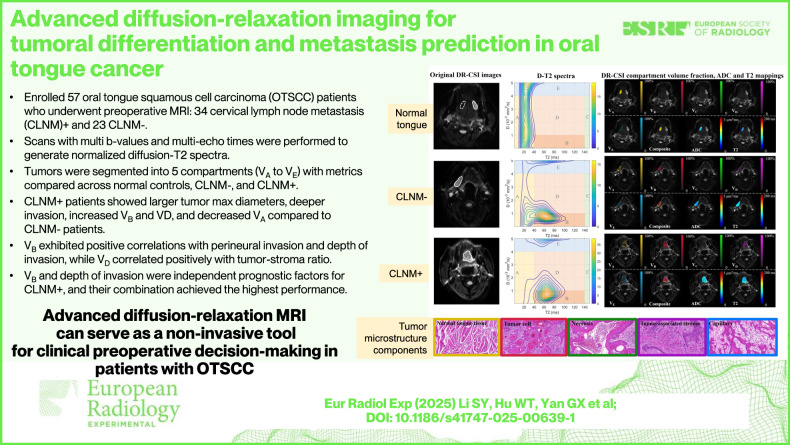

## Introduction

Oral tongue squamous cell carcinoma (OTSCC) represents the most common malignancy of the oral cavity and poses considerable challenges for prognosis [1]. The incidence of OTSCC continues to rise globally, particularly among women and younger individuals [2, 3]. Despite advancements in treatment, including surgery, radiotherapy, and chemotherapy, the 5-year overall survival rate remains below 60% [4]. Approximately 40% of patients will experience recurrence within two years, primarily due to potential local invasiveness and cervical lymph node metastasis (CLNM) [5, 6].

CLNM plays a critical role in determining the prognosis of OTSCC, and the tumor microenvironment profoundly influences its development. The rich lympho-vascular network in the oral tongue facilitates metastasis, making accurate preoperative assessment of CLNM status crucial for effective staging, treatment planning, and improving patient outcomes [7]. Factors such as the depth of invasion (DOI), histological grade, and tumor-stroma ratio (TSR) closely correlate with CLNM [8, 9]. Specifically, a lower TSR, which indicates a higher proportion of stroma relative to tumor cells, correlates with poorer outcomes and an elevated risk of metastasis [10, 11]. Early intervention for CLNM can improve the 5-year disease-free survival rate from 50.7% to 85.1%, highlighting the necessity for accurate detection of CLNM [6].

Existing imaging techniques, including computed tomography and magnetic resonance imaging (MRI), face challenges in accurately distinguishing between benign and malignant lymph nodes [12]. Enlarged reactive lymph nodes or necrotic suppurative lymph nodes can mimic metastatic nodes due to morphological similarities, while metastatic lymph nodes may appear normal in size or signal [13]. Additionally, approximately 30% of early-stage OTSCC cases involve occult lymph node metastasis without radiological evidence [14]. Conventional imaging metrics, such as apparent diffusion coefficient (ADC) and T2 relaxation time value, provide some information on tumor microstructure but are inherently limited [15, 16]. These methods rely on single-parameter assessments and simplified models, often leading to voxel averaging effects that obscure critical intravoxel heterogeneity. Thus, advanced imaging approaches capable of providing a detailed assessment of tumoral profile are warranted to overcome these limitations.

Diffusion-relaxation correlation spectroscopic imaging (DR-CSI) has emerged as a promising technique to address these limitations. Unlike conventional methods, DR-CSI employs a two-dimensional approach that simultaneously resolves diffusion and relaxation properties within a voxel, providing a more nuanced view of tumor microstructure [17]. By disentangling coupled diffusion-relaxation signals, DR-CSI can effectively characterize different tissue compartments, thereby offering novel insights into tumor heterogeneity. Studies have applied DR-CSI in both *in vivo* and *ex vivo* contexts across various tumor types, demonstrating its ability to differentiate cancerous from benign tissues and assess tissue composition at a subvoxel scale [18–21].

In this preliminary study, we hypothesized that DR-CSI could characterize microstructural features with different diffusion and T2 relaxation properties in both benign and malignant tissues of the oral tongue. We aimed to evaluate the feasibility of DR-CSI in characterizing tumor heterogeneity and to assess its potential for predicting CLNM in patients with OTSCC.

## Materials and methods

### Study population

**Fig. 1:**
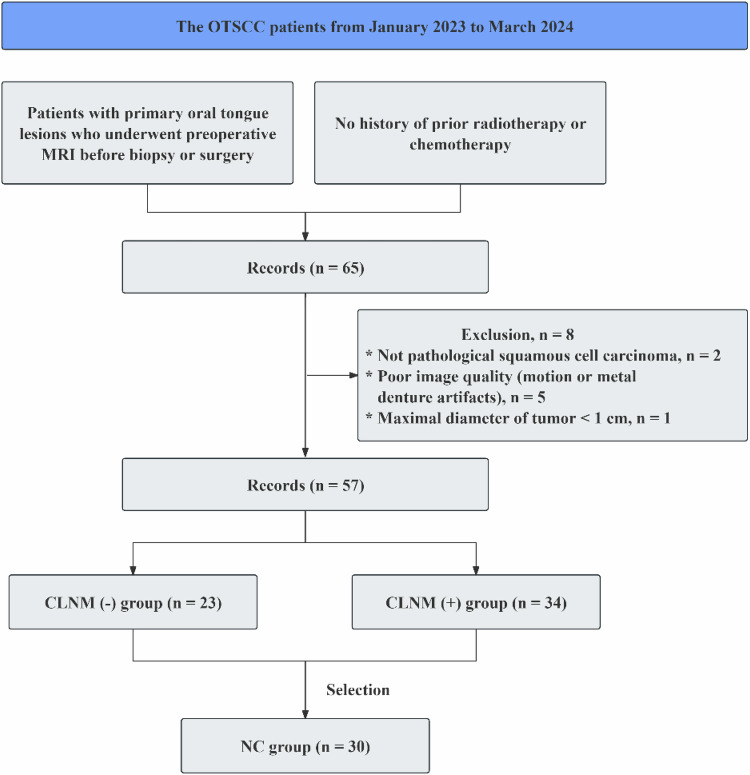
Workflow of inclusion and exclusion of patients. CLNM, Cervical lymph node metastasis; MRI, Magnetic resonance imaging; NC, Normal control; OTSCC, Oral tongue squamous cell carcinoma

This ongoing prospective study was approved by the ethical review board of our institute (No. SH9H-2023-T454-1). Informed consent was obtained from all individuals included in the study. We enrolled consecutive newly diagnosed patients with pathologically proven OTSCC between January 2023 and March 2024. The inclusion criteria were: (1) patients with primary oral tongue lesions who underwent preoperative MRI examinations before biopsy or surgery; (2) no history of prior radiotherapy or chemotherapy. The exclusion criteria were: (1) the lesions were not identified as squamous cell carcinoma on pathology; (2) poor image quality caused by motion or metal denture artifacts; (3) maximal diameter (MD) of tumor < 1 cm. Normal controls (NCs) were selected from those patients with a tumor confined to the midline in this study (Fig. 1).

CLNM status was assessed by preoperative MRI and proven by postoperative pathology. For patients who did not undergo neck dissection or initially presented with a negative CLNM status, clinical and radiological follow-ups were conducted to confirm the status until the last visit.

### MRI acquisition

All patients underwent the MRI examination on a 3.0 T scanner (Magnetom Skyra, Siemens Healthineers) with a commercial 32-channel head and neck coil. The MRI protocols included: axial T1-weighted imaging (repletion time 410 ms, echo time 11 ms; slice thickness/gap 4/0.4 mm/mm; field of view 205 × 220 mm^2^, matrix 540 × 576); axial T2-weighted imaging with fat suppression (2,800 ms, 92 ms; 4/0.4 mm/mm, 205 × 220 mm^2^, 240 × 256); diffusion-weighted imaging (4,600 ms, 58 ms; 4/0.4 mm/mm; 220 × 220 mm^2^; 320 × 320). After injection of the gadopentetate dimeglumine (Gd-DTPA, Magnevist, Bayer AGy), axial, coronal, and sagittal contrast-enhanced T1-weighted sequences were conducted. The contrast agent was injected by a power injector with a dosage of 0.1 mmol/kg at the injection rate of 3 mL/s.

DR-CSI was performed prior to contrast injection using an echo-planar imaging sequence with 35 acquisitions across five echo times (77, 90, 110, 130, and 150 ms), combined with 7 *b*-values (0, 50, 100, 200, 400, 800, 1,500 s/mm²), as described in a previous study [22]. The protocol consisted of 5 successive echo-planar imaging scans, each using a different echo time but the same set of 7 *b*-values. Additional protocol parameters were: repetition time 2,100 ms; slice thickness/gap 4/1.2 mm/mm, field of view 245 × 245 mm^2^, matrix 384 × 384. The scan time for DR-CSI was 5:35 min:s, with the total MRI examination time lasting nearly 30 min per patient.

### Imaging analysis and post-processing

For cases of OTSCC, MD was determined using the largest dimension of the tumor on axial, coronal, and sagittal contrast-enhanced T1-weighted images. DOI was measured on axial contrast-enhanced T1-weighted images by drawing a perpendicular line from the basement membrane of the adjacent intact mucosa to the deepest point of invasion [23]. Regions of interest were manually delineated on the DR-CSI images (echo time = 77 ms, *b*-value = 800 s/mm^2^) using ITK-SNAP software (Penn Image Computing and Science Laboratory, version 4.0.0), covering all slices of the solid tumor. Fat-saturated T2-weighted and contrast-enhanced T1-weighted images were reviewed to exclude necrotic areas. For the NC group, regions of interest of similar sizes were placed on normal tongue tissue contralateral to the tumor. Two experienced head and neck radiologists (S.L. and G.Y.) performed a blinded consensus review of the images, and the interobserver agreement was calculated for MRI metrics.

DR-CSI is a two-dimensional correlation technique that measures coupled voxel-wise signal attenuation caused by both diffusion and relaxation time. The DR-CSI signal could be characterized with the following equation

$${s}_{i}\left(b,\,{T}_{E}\right)={\sum}_{j}{\sum}_{k}{f}_{i}\left({D}_{j},{T}_{2}^{k}\right)* {e}^{-b{D}_{j}}{* e}^{-\frac{{T}_{E}}{{T}_{2}^{k}}}.$$

where $i$ represents a specific voxel, and $j$ and $k$ are the indexes for discrete $T2$ and $D$ in the spectrum, respectively. Each voxel has its unique spectrum, ${f}_{i}\left({D}_{j},{T}_{2}^{k}\right)$, which reveals compositions within a voxel by reflecting different volume fractions. An in-house developed code package based on the MERA fitting tool (version 2.06, https://github.com/markdoes/MERA) was used to fit the spectrum.

Each spectrum was defined using a 30 $D$ * 30 $T2$ mesh grid, spanning the range of $D$: 0.01–5 μm^2^/ms and $T2$: 1–150 ms. The normalized spectra were divided into five subregions: compartments A, B, C, D, and E, respectively. The volume fraction ${V}_{i}$ ($i$ = A, B, C, D, E) of each compartment was calculated for each voxel through spectral integration and then averaged across each patient.

### ADC and T2 mapping

Conventional ADC and T2 mappings were derived from the same DR-CSI data. Specifically, ADC was estimated by linear fitting of signals at *b* = 0/800 s/mm^2^ with a fixed TE of 77 ms, while the T2 value was obtained similarly from data at different echo times with a fixed *b*-value of 0 s/mm². The fitting process was performed using MATLAB (version R2017b; The MathWorks Inc.).

### Histopathology evaluation

After biopsy or surgery, the histological specimens were stained with hematoxylin and eosin and processed for immunohistochemistry. The slices were digitized (Pannoramic microscope scanner, 3DHISTECH Ltd.). All pathological evaluations were initially reported by one pathologist (X.Z., with six years of experience in oral pathology) in a blinded manner and approved by a senior pathologist (Y.H., with ten years of experience in oral pathology). Histological grade, based on the 5th edition of the World Health Organization classification of head and neck tumors [24], was recorded. Pathological parameters, including TSR and Ki-67, were evaluated semiquantitatively, while perineural invasion (PNI), p53 protein, and cyclin-dependent kinase inhibitor p16 were qualified as either positive or negative. TSR was classified as stroma-poor (< 50% stroma) and as stroma-rich (≥ 50% stroma), assessed at the tumor invasive front and calculated as the area of stroma divided by the total area of stroma and tumor combined [25]. PNI was characterized as positive when tumor cells infiltrated any layer of the perineural membrane or grew along a nerve, with involvement exceeding one-third of the nerve circumference [26]. The Ki-67 expression was estimated as the number of stained nuclei divided by all nuclei and was categorized as low or high using a 30% cutoff [27]. The p53 positivity was seen as a nuclear brown stain with varying intensity [28]; p16 expression was performed to determine human papillomavirus positivity.

### Statistical analysis

All statistical analyses were performed in SPSS (version 26.0; IBM Corp.). The continuous variables were presented as mean ± standard deviation, while the categorical variables were described as frequencies and percentage values. Intraclass correlation coefficients were calculated to evaluate interobserver agreement for each MRI metric and interpreted as follows: poor, < 0.4; fair, 0.4–0.59; good, 0.6–0.75; excellent, > 0.75 [29]. The Shapiro-Wilk test was used to test the data normality. We compared the differences of clinical, pathological, and MRI metrics of each of the two groups using an independent *t*-test, Mann–Whitney *U*-test, or *χ*^2^ as appropriate. Univariate and multivariate logistic regression analyses were performed to identify independent predictors of CLNM+, with odds ratios and 95% confidence intervals reported. Multicollinearity among the independent variables was assessed using the variance inflation factor in the linear regression model. Pearson correlation coefficient ($\rho$), Point-biserial correlation coefficient ($\rho$), and Phi coefficient ($\varphi$) were used to determine the relationship between MRI metrics and pathological parameters.

Receiver operating characteristic analysis was conducted to evaluate the prognostic performance of significant metrics and the combined model in predicting CLNM+ status. We incorporated the identified predictors into a nomogram model to assess the risk of CLNM using “R” software (version 4.3.3). Sample size calculation was performed using PASS software (version 15, NCSS, LLC., Kaysville, Utah, USA). Based on this calculation, a sample size of 57 patients was determined to provide 82% statistical power for the study, assuming a baseline probability of CLNM of 0.5, a coefficient of determination (*R*²) of 0.6 for covariate adjustment, and using a two-tailed hypothesis test at a significance level of 0.05. A *p*-value < 0.05 was considered statistically significant.

## Results

### Clinical data

Fifty-seven patients with OTSCC were enrolled in the current study. The average follow-up time to access CLNM status was 6.2 ± 3.0 months. The clinicopathological characteristics of the included patients are summarized in Table 1. All patients tested negative for human papillomavirus expression. There were no significant differences in age or gender between the CLNM+ and CLNM- groups. However, the CLNM+ group had significantly larger MDs and DOIs compared to the CLNM- group. Additionally, advanced cT stages and positive PNIs were more prevalent in the CLNM+ group. Although a trend toward higher histological grades was observed in the CLNM + group, the difference was not significant.

**Table 1:** Clinical and pathological characteristics of patients

	Total (*n* = 57)	CLNM- (*n* = 23)	CLNM + (*n* = 34)	*p-*value
Age (years)	54.2 ± 15.1	55.3 ± 14.3	53.5 ± 15.9	0.663
Sex (*n*, %)				0.599
Male	37 (64.9)	14 (60.9)	23 (67.6)	
Female	20 (35.1)	9 (39.1)	11 (32.4)	
cT Stage (*n*, %)				**0.015**
T1 or T2	17 (29.8)	11 (47.8)	6 (17.6)	
T3 or T4	40 (70.2)	12 (52.2)	28 (82.4)	
MD (cm)	3.1 ± 1.3	2.7 ± 1.0	3.4 ± 1.4	**0.029**
DOI (cm)	1.5 ± 0.9	1.1 ± 0.5	1.8 ± 1.0	**0.002**
HG (*n*, %)				0.189
GI / I–II	39 (68.4)	18 (78.3)	21 (61.8)	
GII / II–III / III	18 (31.6)	5 (21.7)	13 (38.2)	
TSR (*n*, %)				0.242
Stroma-rich	20 (35.1)	6 (26.1)	14 (41.2)	
Stroma-poor	37 (64.9)	17 (73.9)	20 (58.8)	
PNI (*n*, %)				**0.018**
Negative	24 (42.1)	14 (60.9)	10 (29.4)	
Positive	33 (57.9)	9 (39.1)	24 (70.6)	
Ki-67 (*n*, %)				0.093
Low	30 (52.6)	9 (39.1)	21 (61.8)	
High	27 (47.4)	14 (60.9)	13 (38.2)	
p53 (*n*, %)				0.768
Negative	21 (36.8)	9 (39.1)	12 (35.3)	
Positive	36 (63.2)	14 (60.9)	22 (64.7)	

Bold denotes statistically significant results (*p* <  0.05)

*CLNM* Cervical lymph node metastasis, *cT* Clinical T stage, *DOI* Depth of invasion, *HG* Histological grade, *MD* Maximal diameter, *PNI* Perineural invasion, *TSR* tumor-stroma ratio

### DR-CSI spectra segmentation among NC, CLNM-, and CLNM+ groups

**Fig. 2:**
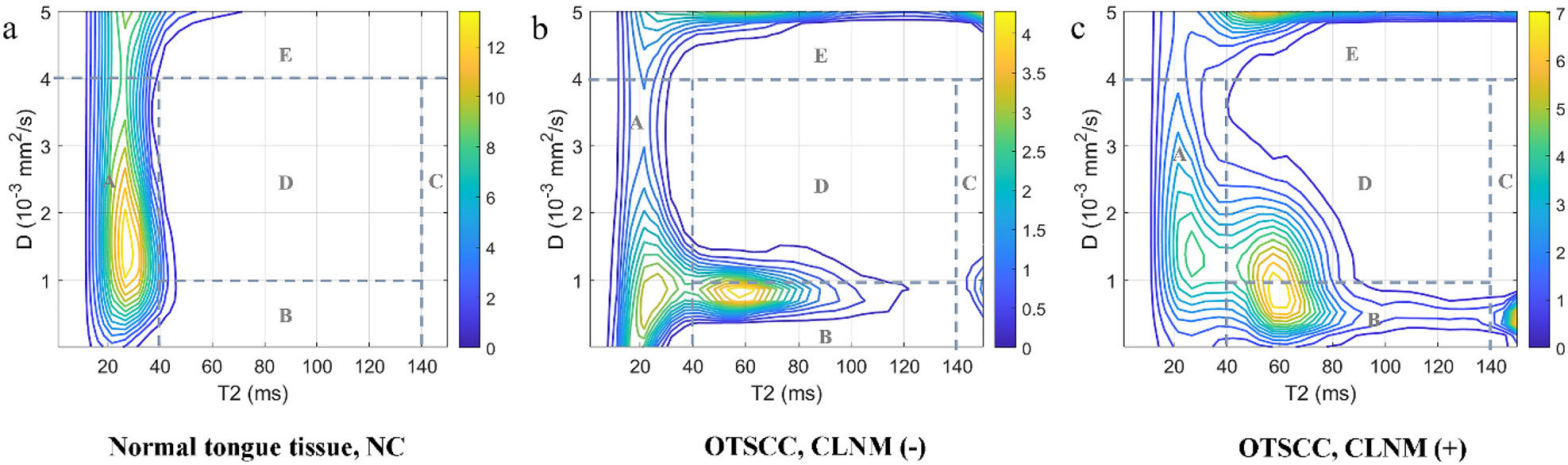
Spectra segmentation strategy. Typical spectra of: **a** normal tongue tissue from a 54-year-old female, NC; **b** a 62-year-old male with OTSCC, CLNM-; **c** a 63-year-old male with OTSCC, CLNM+. The spectra were divided by dashed lines into five compartments to illustrate different microstructures suggested in this study. CLNM, Cervical lymph node metastasis; NC, Normal control; OTSCC, Oral tongue squamous cell carcinoma

Based on previous studies, the spectra were generally segmented according to the distribution of the spectral peaks [18, 22]. The representative DR-CSI spectral segmentation is illustrated in Fig. 2. In normal tongue tissue, two distinct peaks were observed: compartment A with short T2 (< 40 ms) and low-medium diffusivity (< 4 μm²/ms), and compartment E with high diffusivity (> 4 μm²/ms). In OTSCC, additional compartments emerged: compartment B with intermediate T2 (40–140 ms) and low diffusivity (< 1 μm^2^/ms) and compartment D with intermediate T2 (40–140 ms) and intermediate diffusivity (1–4 μm^2^/ms). Notably, the peaks in compartment C, with long T2 (> 140 ms) and low-intermediate diffusivity (< 4 μm^2^/ms) were exclusively observed in OTSCC cases.

**Fig. 3:**
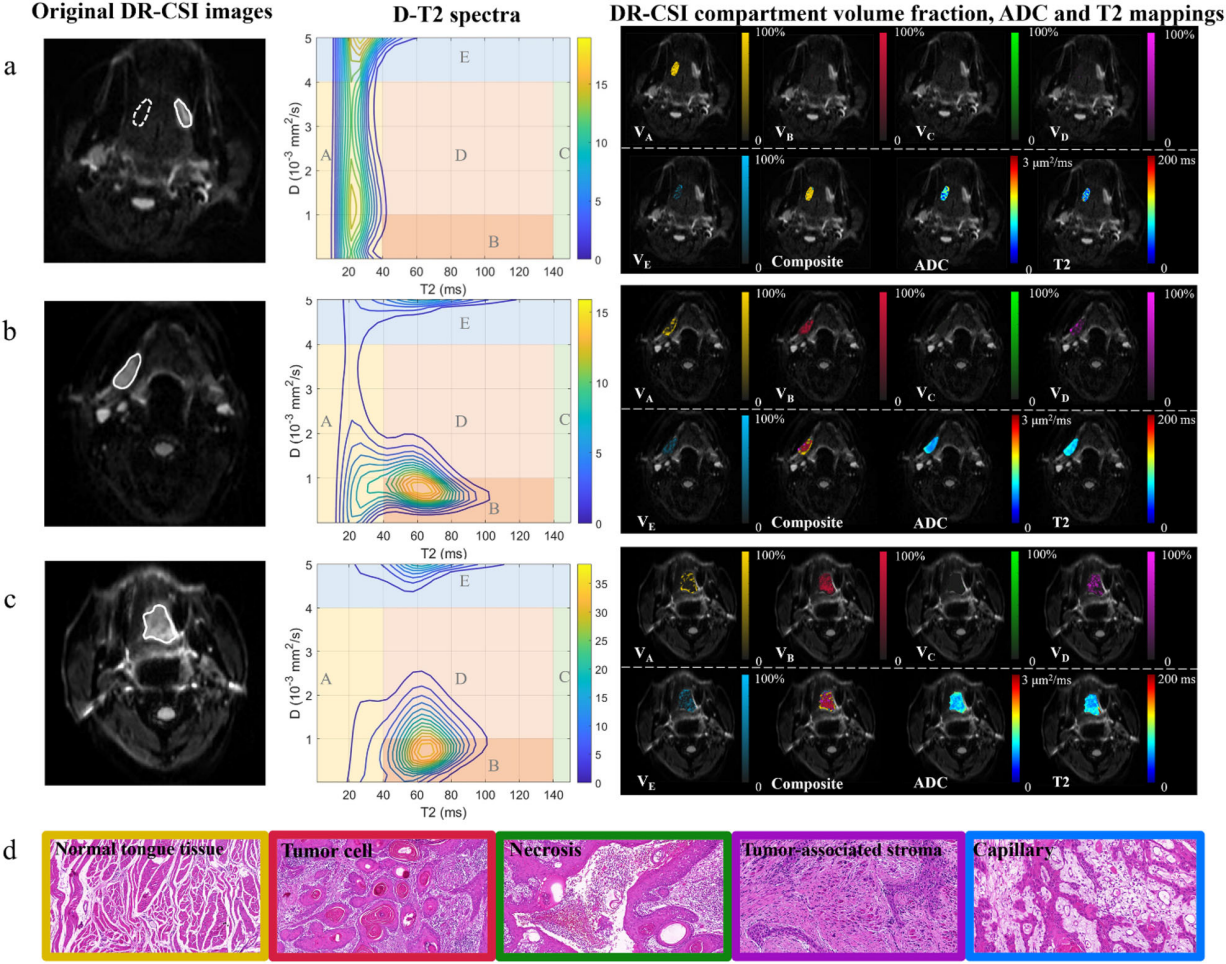
Typical cases from NC, OTSCC with CLNM- and CLNM+ groups. Original DR-CSI images (*b* = 800 s/mm^2^, echo time = 77 ms), Diffusion-T2 spectra and DR-CSI volume fraction mappings, ADC and T2 mappings of ROIs of: **a** normal tongue tissue from a 50-year-old female with OTSCC; **b** a 72-year-old male with OTSCC, CLNM-; **c** a 63-year-old male with OTSCC, CLNM+. The ROIs of tumors were labeled in a solid white circle, while the contralateral normal tongue tissue was labeled in a dashed white circle; **d** a schematic diagram associating DR-CSI *V*_A_-*V*_E_ with tumor microstructure components. *V*_A_ (yellow): normal tongue tissue, *V*_B_ (blue): tumor cell, *V*_C_ (green): necrosis, *V*_D_ (purple): tumor-associated stroma, *V*_E_ (blue): capillary. ADC, Apparent diffusion coefficient; CLNM, Cervical lymph node metastasis; DR-CSI, Diffusion-relaxation correlation spectroscopic imaging; NC, Normal control; ROIs, Regions of interest; OTSCC, Oral tongue squamous cell carcinoma

Figure 3 illustrates the original DR-CSI images, diffusion-T2 spectra, and corresponding spatial volume fraction, ADC, and T2 mapping for three representative cases, highlighting the compartmental changes with the development and progression of OTSCC. In normal tongue tissue, *V*_A_ was the predominant compartment. With the onset of OTSCC, *V*_A_ decreased significantly, while *V*_B_ and *V*_D_ increased. In CLNM+ cases, *V*_B_ and *V*_D_ showed further prominent development, with *V*_B_ dominating the tumor invasive front, accompanied by an even greater reduction in *V*_A_. The schematic diagram explicitly mapped each compartment to the associated tumor microstructure component.

### Comparison of MRI and pathological metrics among NC, CLNM-, and CLNM+ groups

Interobserver agreement was excellent for MRI metrics (intraclass correlation coefficient: 0.874–0.986), as summarized in Table S1. The DR-CSI volume fractions, ADC, and T2 values across NC, CLNM-, and CLNM+ groups are summarized in Table 2 and illustrated in Supplemental Fig. S1. Significant differences in *V*_A_, *V*_B_, *V*_D_, and ADC were observed between each pair of two groups (*p*-values from 0.003 to 0.001). Notably, decreased *V*_A_ and ADC were detected in OTSCC being CLNM- compared with normal tongue tissue (*V*_A_: 75.6 ± 8.0% *versus* 47.9 ± 20.8%, *p* < 0.001; ADC: 1.47 ± 0.16 *versus* 1.07 ± 0.17 μm^2^/ms, *p* < 0.001); lower value was seen in comparison with CLNM+ (*V*_A_: 47.9 ± 20.8%, *versus* 30.3 ± 14.8%, *p* < 0.001; ADC: 1.07 ± 0.17 *versus* 0.96 ± 0.09 μm^2^/ms, *p* = 0.021). In contrast, *V*_B_ and *V*_D_ continuously increased with the OTSCC progression, reaching the highest values in the CLNM+ group. *V*_C_, *V*_E_, and T2 values differed between CLNM- and CLNM+ groups but were not statistically different.

**Table 2:** Comparison of DR-CSI metrics, ADC, and T2 values among NC, CLNM-, and CLNM+ groups

Metrics	NC (*n* = 30)	CLNM- (*n* = 23)	CLNM+ (*n* = 34)	*p*-value^a^	*p*-value^b^	*p*-value^c^
*V*_A_ (%)	75.6 ± 8.0	47.9 ± 20.8	30.3 ± 14.8	**<** **0.001**	**<** **0.001**	**<** **0.001**
*V*_B_ (%)	1.4 ± 1.4	17.4 ± 11.8	28.9 ± 10.9	**<** **0.001**	**<** **0.001**	**<** **0.001**
*V*_C_ (%)	0.1 ± 0.2	0.4 ± 0.5	0.6 ± 0.7	**0.009**	**0.001**	0.591
*V*_D_ (%)	2.6 ± 3.5	16.2 ± 10.0	24.6 ± 10.0	**<** **0.001**	**<** **0.001**	**0.003**
*V*_E_ (%)	20.4 ± 7.5	18.0 ± 10.3	15.5 ± 5.5	0.099	**0.004**	0.757
ADC (μm^2^/ms)	1.47 ± 0.16	1.07 ± 0.17	0.96 ± 0.09	**<** **0.001**	**<** **0.001**	**0.021**
T2 (ms)	52.6 ± 11.9	60.0 ± 15.0	61.8 ± 16.0	**0.024**	**0.002**	0.637

Independent *t-*test and Mann–Whitney *U*-test were used to compare the difference in metrics between each pair of groups

Bold denotes statistically significant results (*p*  <  0.05)

*ADC* Apparent diffusion coefficient, *CLNM* Cervical lymph node metastasis, *DR-CSI* Diffusion-relaxation correlation spectroscopic imaging

^a^ NC *versus* CLNM-

^b^ NC *versus* CLNM+

^c^ CLNM- *versus* CLNM+

**Fig. 4:**
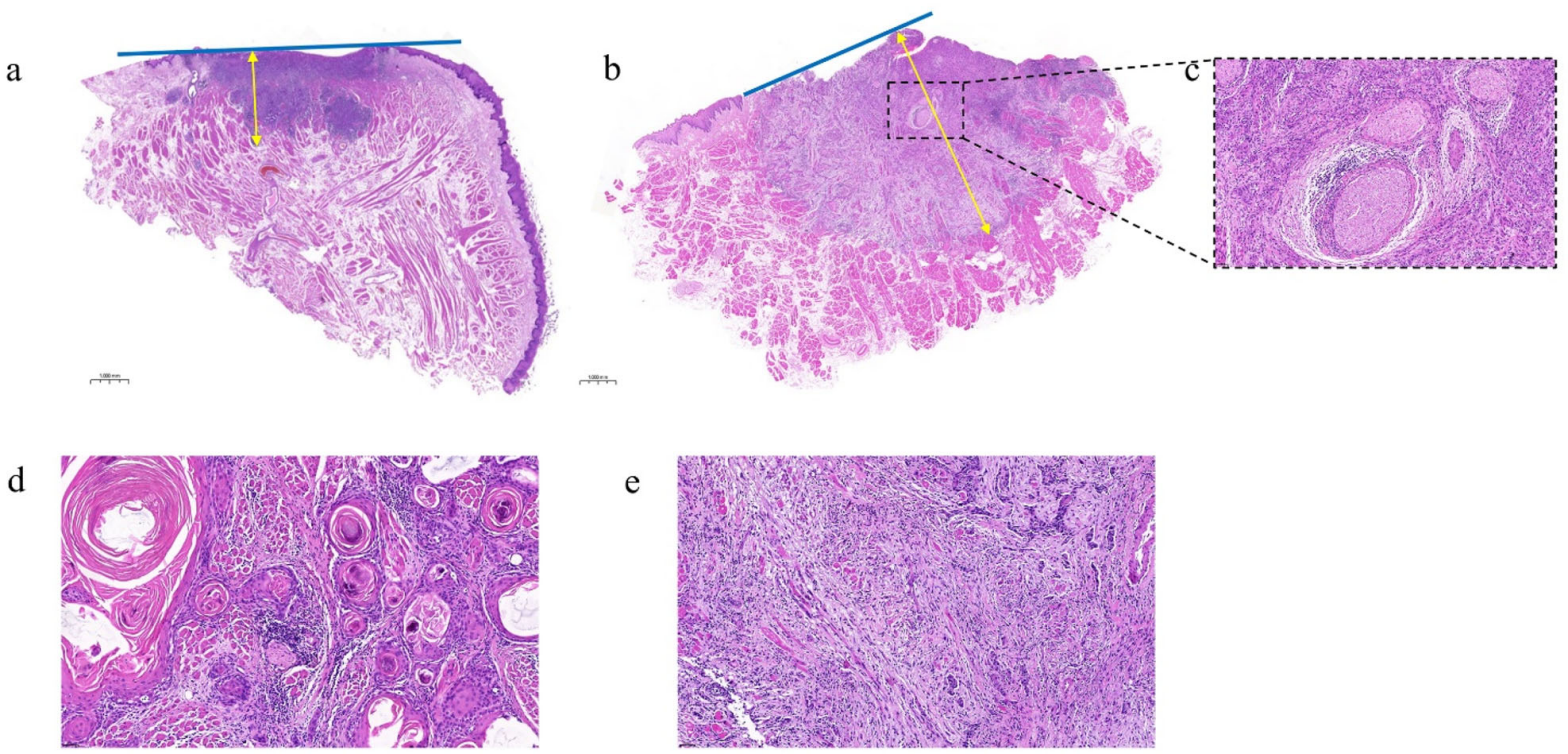
H&E staining slices of OTSCC. Measurement of DOI (yellow arrow, ×1). **a** CLNM-, DOI < 5 mm; **b** CLNM+, 5 < DOI < 10 mm; **c** PNI+ (×20). Assessment of TSR at TIF (×20); **d** CLNM-, stroma-poor; **e** CLNM+, stroma-rich. CLNM, Cervical lymph node metastasis; DOI, Depth of invasion; H&E, Hematoxylin and eosin; OTSCC, Oral tongue squamous cell carcinoma; PNI, Perineural invasion; TIF, Tumor invasive front; TSR, Tumor-stroma ratio

Figure 4 shows the pathological features of DOI, PNI, and TSR on hematoxylin and eosin staining sections. The CLNM+ case displayed a deeper DOI and showed PNI+. In the tumor invasive front, the CLNM+ case exhibited stroma-rich characteristics, whereas the CLNM- case was stroma-poor.

### Predictive modeling of CLNM+

**Fig. 5:**
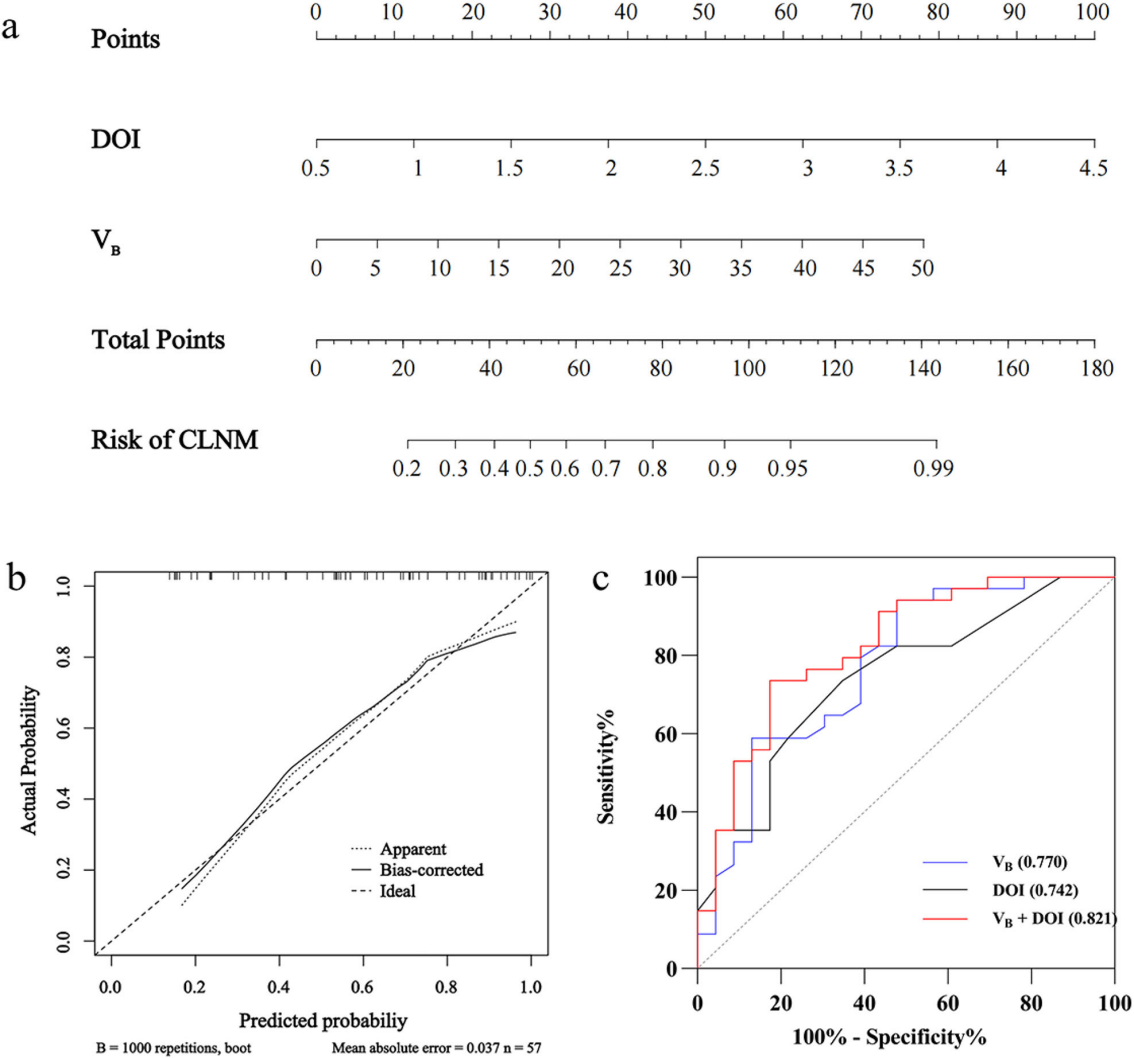
**a** Nomogram model for estimating the risk of CLNM in OTSCC. **b** Calibration curve for the nomogram. **c** ROC curves for single metrics and the combined model in predicting CLNM. CLNM, Cervical lymph node metastasis; DOI, Depth of invasion; OTSCC, Oral tongue squamous cell carcinoma; ROC, receiver operating characteristic

Univariate logistic regression analysis identified that cT stage (*p* = 0.018), MD (*p* = 0.043), DOI (*p* = 0.011), *V*_A_ (*p* = 0.002), *V*_B_ (*p* = 0.002), *V*_D_ (*p* = 0.006), ADC (*p* = 0.012), and PNI (*p* = 0.021) were associated with CLNM+ status. Further multivariate logistic regression identified *V*_B_ (odds ratio, 1.079; confidence interval: 1.018–1.145; *p* = 0.011) and DOI (odds ratio, 3.394; confidence interval: 1.015–11.348; *p* = 0.047) as independent predictors for CLNM (Table 3). The variance inflation factor for *V*_B_ and DOI was 1.106, suggesting no multicollinearity concerns. Figure 5 exhibits the nomogram model constructed based on *V*_B_ and DOI. The calibration curve was close to the ideal performance, which indicated the predictive accuracy of the nomogram.

**Table 3:** Univariate and multivariate logistic regression analyses of variables associated with CLNM in OTSCC patients

	OR (95% CI)	*p*-value
**Univariate analysis**		
Age (years)	0.992 (0.957, 1.028)	0.657
Sex (Female, %)	0.744 (0.247, 2.243)	0.599
cT Stage (T3-4, %)	4.278 (1.285, 14.243)	**0.018**
MD (cm)	1.734 (1.018, 2.953)	**0.043**
DOI (cm)	4.539 (1.408, 14.634)	**0.011**
*V*_A_ (%)	0.946 (0.913, 0.980)	**0.002**
*V*_B_ (%)	1.096 (1.035, 1.161)	**0.002**
*V*_C_ (%)	1.670 (0.632, 4.414)	0.301
*V*_D_ (%)	1.088 (1.024, 1.155)	**0.006**
*V*_E_ (%)	0.958 (0.890, 1.031)	0.250
ADC (μm^2^/ms)	0.002 (0.000, 0.244)	**0.012**
T2 (ms)	1.008 (0.973, 1.043)	0.665
HG (GII / II–III / III,%)	2.229 (0.666, 7.461)	0.194
TSR (Stroma-rich, %)	1.983 (0.625, 6.291)	0.245
Ki-67 (High, %)	0.398 (0.134, 1.179)	0.096
PNI (Positive, %)	3.733 (1.223, 11.396)	**0.021**
p53 (Positive, %)	1.179 (0.395, 3.518)	0.786
**Multivariate analysis**		
DOI (cm)	3.394 (1.015, 11.348)	**0.047**
*V*_B_ (%)	1.079 (1.018, 1.145)	**0.011**

Bold denotes statistically significant results (*p*  <  0.05)

*ADC* Apparent diffusion coefficient, *CI* Confidence interval, *CLNM* Cervical lymph node metastasis, *cT* Clinical T stage, *DOI* Depth of invasion, *HG* Histological grade, *MD* Maximal diameter, *OR* Odds ratio, *OTSCC* Oral tongue squamous cell carcinoma, *PNI* Perineural invasion, *TSR* Tumor-stroma ratio

The receiver operating characteristic analysis of the MRI metrics found to be statistically significant in logistic regression analysis are shown in Table 4 and Supplemental Fig. S2. Therein, *V*_B_ demonstrated the highest predictive performance for CLNM, with an area under the curve (AUC) of 0.770 (sensitivity 0.941, specificity 0.522). DOI showed strong predictive ability (AUC 0.742, sensitivity 0.735; specificity 0.652), outperforming ADC (AUC 0.681, sensitivity 0.912, specificity 0.435) and MD (AUC 0.672, sensitivity 0.471, specificity 0.870). Notably, the predictive model combining DOI and *V*_B_ yielded the highest AUC of 0.821 (sensitivity 0.735; specificity 0.826), demonstrating the synergistic value of these metrics in assessing CLNM status.

**Table 4:** Predictive performance of MRI metrics and the combined model

	AUC (95% CI)	Sensitivity	Specificity	Cutoff
*V*_A_	0.749 (0.615, 0.883)	0.529	0.913	25.6%
*V*_B_	0.770 (0.641, 0.898)	0.941	0.522	13.0%
*V*_D_	0.728 (0.593, 0.862)	0.824	0.391	16.6%
ADC	0.681 (0.538, 0.824)	0.912	0.435	1.10 μm^2^/ms
MD	0.672 (0.531, 0.813)	0.471	0.870	3.5 cm
DOI	0.742 (0.612, 0.871)	0.735	0.652	1.2 cm
*V*_B_ + DOI	0.821 (0.708, 0.933)	0.735	0.826	

*ADC* Apparent diffusion coefficient, *AUC* Area under the curve, *DOI* Depth of invasion, *MD* Maximal diameter

### Correlation between MRI metrics and pathological biomarkers

**Fig. 6:**
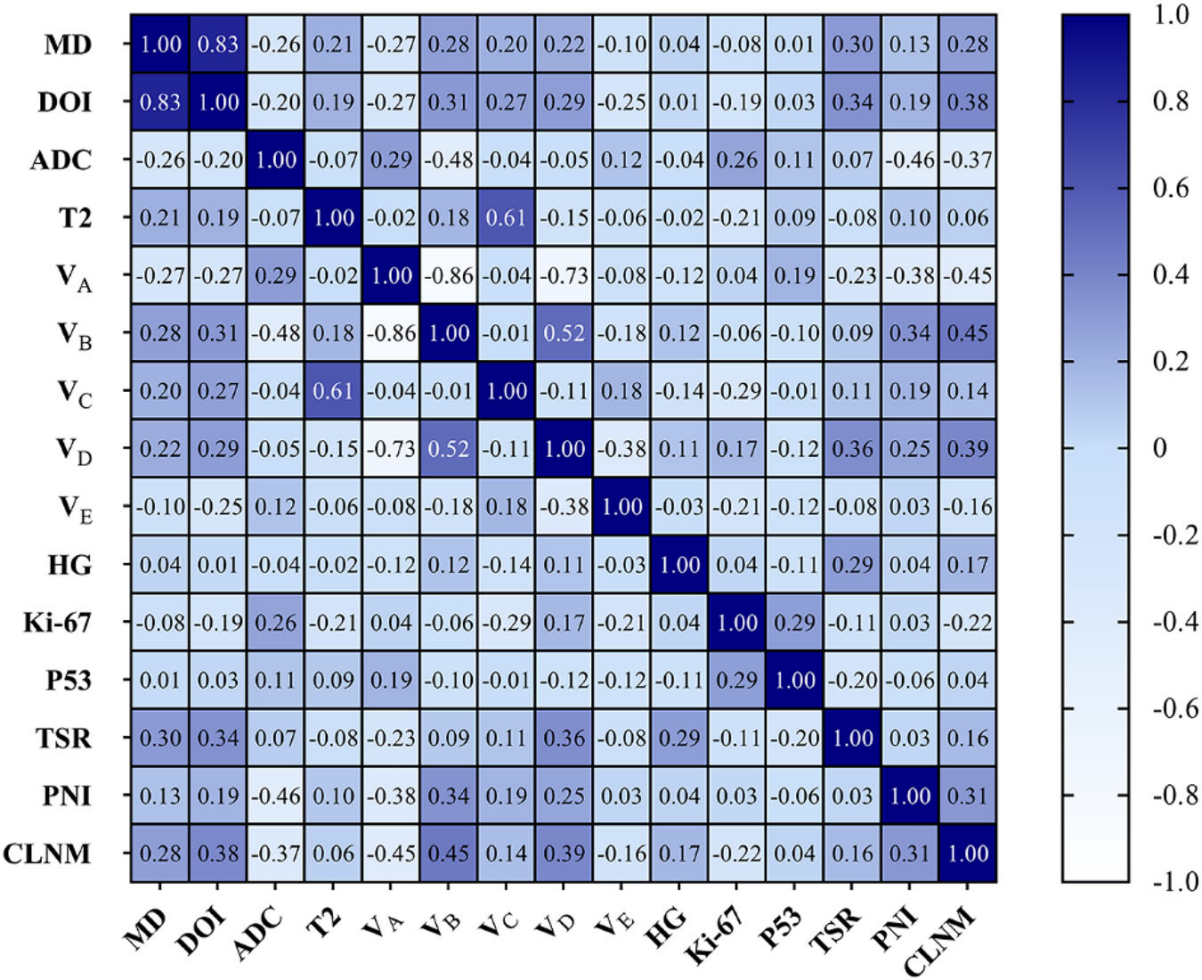
Correlation between MRI metrics and pathological biomarkers. *ADC,* Apparent diffusion coefficient; CLNM, Cervical lymph node metastasis; DOI, Depth of invasion; HG, Histological grade; MD, Maximal diameter; PNI, Perineural invasion; TSR, Tumor-stroma ratio

The relationships between MRI-derived metrics and pathological biomarkers were evaluated, as depicted in Fig. 6. *V*_A_, predominantly found in normal tongue tissue, demonstrated negative correlations with aggressive pathological markers, including TSR, PNI, and positive CLNM ($\rho$ = -0.23, -0.38, and -0.45, respectively). In contrast, *V*_B_ exhibited significant positive correlations with aggressive tumor features, such as PNI and positive CLNM ($\rho$ = 0.34, 0.45, respectively), suggesting that *V*_B_ may serve as a potential biomarker of tumor invasiveness. *V*_D_ positively correlated with TSR ($\rho$ = 0.36), indicating its potential as an indicator of tumor-associated stroma. Strong interrelationships were observed among DR-CSI metrics. For instance, *V*_A_ negatively correlated with both *V*_B_ ($\rho$ = -0.86) and *V*_D_ ($\rho$ = -0.73), suggesting these metrics captured distinct and opposing features of tumor composition. In addition, tumor size metrics, such as MD and DOI, showed positive correlations with both *V*_B_ and *V*_D_, further linking these metrics to tumor progression.

## Discussion

Our study highlights the potential of DR-CSI in advancing the diagnostic and prognostic assessment of OTSCC. By segmenting diffusion-T2 spectra into five distinct compartments (from *V*_A_ to *V*_E_), we were able to differentiate tumor tissue from normal tongue tissue and provide a characterization of the tumoral differential profile. These findings offer valuable insights into how DR-CSI metrics relate to CLNM status and other pathological biomarkers.

*V*_A_, dominating normal tongue tissue with short T2 and low-intermediate diffusivity, correlated negatively with invasive pathological biomarkers. The marked reduction of V_A_ in CLNM+ patients indicates progressive tumor invasion into normal tissue and higher malignancy. *V*_B_, with intermediate T2 and low diffusivity, likely reflects invasive tumor cells. The significant increase of *V*_B_ in the CLNM+ group suggests restricted water diffusion, caused by a high cellular density that reduces extracellular space and limits water diffusion. Additionally, poorly differentiated tumors, which are frequently observed in the CLNM+ group, lack keratinization and intercellular bridges, further hindering diffusion [30]. These findings align with previous studies, including that by Ren et al [31], who reported lower ADC_10th_ values in early-stage OTSCC with occult CLNM+. Importantly, the spatial concentration of *V*_B_ at the tumor invasive front and its positive correlations with PNI ($\rho$ = 0.34) and DOI ($\rho$ = 0.31) reinforce *V*_B_ as an indicator of tumor invasiveness.

Alongside tumoral differential profile, we identified the components *V*_C_, *V*_D_, and *V*_E_ as key markers of the tumor microenvironment. *V*_C_, which is characterized by long T2, is likely associated with necrotic regions commonly seen in large or poorly differentiated tumors [32]. Previous DR-CSI studies on renal carcinoma have linked longer T2 compartments to cystic changes [18]. In this study, despite excluding visible necrotic regions, we observed elevated *V*_C_ during OTSCC progression, suggesting that non-visible necrosis may contribute significantly to tumor growth and metastasis. *V*_D_, a medium T2 and medium diffusivity component, has been recognized as a marker for tumor invasion. Previous research has shown higher ADCs in stroma-rich cancers compared to stroma-poor cancers [33, 34]. The correlation between *V*_D_ and the TSR ($\rho$ = 0.36) in our study suggests that *V*_D_ could serve as a marker of tumor-associated stroma. The stroma, an essential part of the tumor microenvironment, plays a crucial role in supporting both normal and malignant epithelial tissues [35]. An increase in *V*_D_ may indicate greater stromal content, creating a more favorable environment for metastasis. *V*_E_, which exhibits high diffusivity, may correspond to capillary perfusion [36]. *V*_E_ was highest in normal tongue tissues but gradually decreased with tumor progression, although the difference did not reach statistical significance. This trend can be attributed to an increasing number of immature and disorganized blood vessels in the tumor [37]. These findings highlight the capability of DR-CSI to disentangle and correlate microstructures with pathological biomarkers, providing a more nuanced understanding of OTSCC invasiveness and metastatic potential.

In our study, *V*_B_ and DOI were identified as independent predictors of CLNM+, underscoring their value in non-invasive assessment. *V*_B_ achieved the highest AUC and sensitivity among univariate variables, reinforcing its superior diagnostic power in characterizing tumor heterogeneity and invasive tumor characteristics. In contrast, the macroscopic properties of ADC, which fail to capture subvoxel heterogeneity, limit its utility as an imaging biomarker for assessing the tumor microenvironment. DR-CSI, however, provides a two-dimensional correlation of diffusion and relaxation properties, enabling it to disentangle contributions from different microstructural components within a voxel. This capability enables DR-CSI to offer more precise insights into tumor heterogeneity compared to mono-exponential models like ADC or bi-exponential models such as intravoxel incoherent motion (IVIM), which fail to capture the complexity of the tumor microenvironment [38].

Our results indicated that while T2 values were slightly higher in the CLNM+ group, the difference was not statistically significant. The diffusion-T2 spectra revealed variations across different spans, each corresponding to a specific compartment. This can be attributed to the averaging effect of heterogeneous subvoxel components in conventional T2WI. Although T2 texture analysis has been used to predict occult lymph node metastasis in early-stage OTSCC with an AUC of 0.739 [14], it still lacks the ability to directly link imaging metrics to underlying microstructures. In contrast, DR-CSI enhances both data dimensionality and spatial information, providing a more detailed assessment of tumor composition [39].

DR-CSI represents an innovative technique poised for integration into clinical workflows. By simultaneously disentangling both diffusion and relaxation properties, DR-CSI offers a more nuanced assessment of tissue microstructure than conventional MRI methods. Crucially, its capability to deliver detailed insights without exogenous contrast agents positions it as a complementary tool to existing imaging modalities. In oncology, evidence from previous research [18, 20] and this study suggests DR-CSI holds potential for early detection of occult metastasis, monitoring treatment responses, and resolving tumor heterogeneity at a microstructural level. DR-CSI can be effectively integrated into clinical workflows by optimizing post-processing times to ensure they are within acceptable limits. In the current study, post-processing of DR-CSI data was performed on a personal computer using MATLAB software with parallel computation, taking approximately 10 min for a three-layer ROI of a tumor with a mean diameter of 2 cm. While this is slightly longer than conventional diffusion-weighted imaging, ongoing advancements in software and computational techniques are expected to reduce processing times, making it more feasible for routine clinical use. Given the integration of parameters of conventional diffusion-weighted imaging, DR-CSI could either complement diffusion-weighted imaging or, in some cases, replace it, especially when deeper insights into tumor heterogeneity and treatment response are required [40, 41].

This study has several limitations. The primary limitation stems from the asymptomatic nature of OTSCC, which often leads to advanced-stage diagnoses and creates an imbalance between CLNM+ and CLNM- cases in our cohort, with a predominance of advanced-stage (cT3 or cT4) cases. To address these biases and further validate our findings, future multi-center studies with larger cohorts are essential. Additionally, the lack of a double-blinded pathological evaluation may introduce measurement bias. Due to the *in vivo* nature of the study, histological validation for compartmental mapping was not feasible. Future work will involve prospective correlations with histological mapping. Translating DR-CSI into routine clinical practice also faces challenges, including empirical segmentation and result interpretation, both of which require specialized expertise and are prone to inter-institutional variability. While AI-driven segmentation offers promising solutions, it still depends on large, annotated datasets to ensure reliable generalizability. Although the intraclass correlation coefficient results demonstrate good interobserver consistency for all DR-CSI metrics in the current study, larger cohorts and external validation are necessary to confirm reproducibility and establish generalizability. Moreover, the relatively long acquisition time of DR-CSI (approximately 5 minutes) increases the risk of motion artifacts and computational costs, potentially limiting its broader clinical application. Advances in faster imaging techniques are needed to reduce acquisition time and mitigate these challenges.

In conclusion, DR-CSI represents a transformative advancement in imaging for OTSCC. By providing a detailed characterization of tumor component and improving the prediction of CLNM status, DR-CSI metrics such as *V*_B_ and *V*_D_ are able to characterize tumor heterogeneity. The ability to correlate these metrics with key biomarkers makes DR-CSI a promising tool for clinical applications. Thereby, integrating DR-CSI into preoperative decision-making could help reduce unnecessary lymph node dissections in specific patient populations.

## Supplementary information

ELECTRONIC SUPPLEMENTARY MATERIAL

## Data Availability

The datasets analyzed in the current study are not publicly available due to ethical principles. However, the data are available from the corresponding author upon reasonable request.

## Abbreviations

ADC: Apparent diffusion coefficient
AUC: Area under the (receiver operating characteristic) curve
CLNM: Cervical lymph node metastasis
DOI: Depth of invasion
DR-CSI: Diffusion-relaxation correlation spectroscopic imaging
MD: Maximal diameter
MRI: Magnetic resonance imaging
NC: Normal control
OTSCC: Oral tongue squamous cell carcinoma
PNI: Perineural invasion
TSR: Tumor-stroma ratio
