# Supplementary material for: Advanced diffusion-relaxation imaging for tumoral differentiation and metastasis prediction in oral tongue cancer
Source: Eur Radiol Exp. 2025 Oct 8;9:99. doi: 10.1186/s41747-025-00639-1 (PMC12508348; doi:10.1186/s41747-025-00639-1)
Supplement: Supplementary file 1 — ELECTRONIC SUPPLEMENTARY MATERIAL [file 41747_2025_639_MOESM1_ESM.pdf]

Advanced diffusion-relaxation imaging for tumoral differentiation and metastasis prediction in oral tongue cancer

ELECTRONIC SUPPLEMENTARY MATERIAL

Table S1. The ICC analysis for interobserver variability in ROI delineation.

MRI metrics	ICC (95% CI)
V_A (%)	0.964 (0.939, 0.979)
V_B (%)	0.941 (0.900, 0.965)
V_C (%)	0.874 (0.785, 0.926)
V_D (%)	0.909 (0.846, 0947)
V_E (%)	0.899 (0.828, 0.940)
ADC ($\mu m^2/ms$)	0.917 (0.859, 0.951)
T2 (ms)	0.932 (0.885, 0.960)
MD (cm)	0.985 (0.974, 0.991)
DOI (cm)	0.986 (0.977, 0.992)

Note: ADC, apparent diffusion coefficient; CI, confidence interval; DOI, depth of invasion; ICC, intraclass correlation coefficient; MD, maximal diameter; ROI, region of interest.

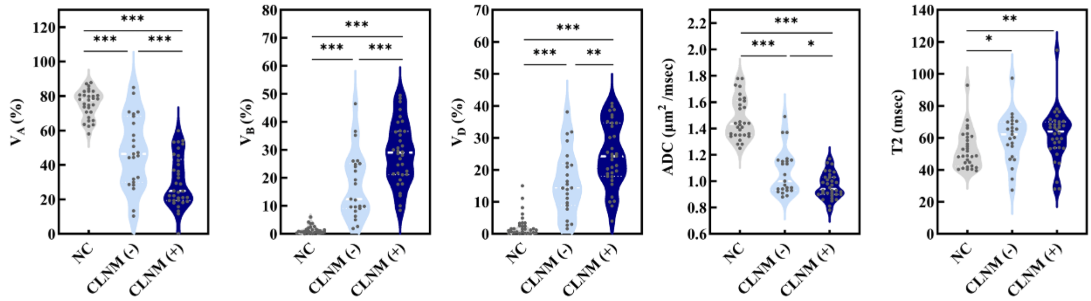

Fig. S1 Comparison of (a) DR-CSI V_A; (b) V_B; (c) V_D; (d) ADC and (e) T2 value among NC, CLNM (-) and CLNM (+) groups, presented as violin plots.

Note: Significant levels: *, $p < 0.05$; **, $p < 0.01$; ***, $p < 0.001$.

ADC, apparent diffusion coefficient; CLNM, cervical lymph node metastasis; DR-CSI, diffusion-relaxation correlation spectroscopic imaging; NC, normal control.

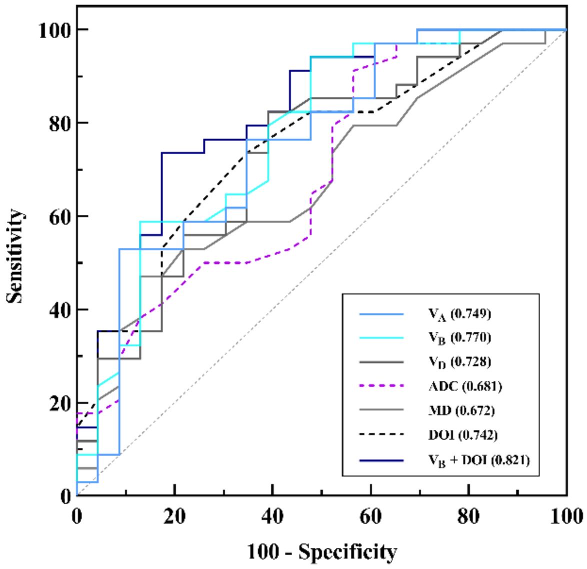

Fig. S2 ROC curves for single MRI metrics and the combined model in predicting CLNM of OTSCC patients.

Note: ADC, apparent diffusion coefficient; CLNM, cervical lymph node metastasis; DOI, depth of invasion; MD, maximal diameter; OTSCC, oral tongue squamous cell carcinoma; ROC, receiver operating characteristic.
